# The Safety and Anti-Tumor Effects of Ozonated Water *in Vivo*

**DOI:** 10.3390/ijms161025108

**Published:** 2015-10-22

**Authors:** Kohei Kuroda, Kazuo Azuma, Takuro Mori, Kinya Kawamoto, Yusuke Murahata, Takeshi Tsuka, Tomohiro Osaki, Norihiko Ito, Tomohiro Imagawa, Fumio Itoh, Yoshiharu Okamoto

**Affiliations:** 1 Department of Clinical Medicine, Joint School of Veterinary Medicine, Faculty of Agriculture, Tottori University, Tottori 680-8553, Japan; E-Mails: nanararayu@gmail.com (K.Ku.); kazu-azuma@muses.tottori-u.ac.jp (K.A.); t_k_low15@yahoo.co.jp (T.M.); kawamotokinya@yahoo.co.jp (K.Ka.); ymurahata@muses.tottori-u.ac.jp (Y.M.); tsuka@muses.tottori-u.ac.jp (T.T.); tosaki@muses.tottori-u.ac.jp (T.O.); taromobile@me.com (N.I.); imagawat@muses.tottori-u.ac.jp (T.I.); 2 Department of Technical Development, Sakuragawa Pump Co., Ltd., Osaka 567-0005, Japan; E-Mail: itoh@ sakura-p.net

**Keywords:** ozone, ozonated water, anti-tumor effect, reactive oxygen species

## Abstract

Ozonated water is easier to handle than ozone gas. However, there have been no previous reports on the biological effects of ozonated water. We conducted a study on the safety of ozonated water and its anti-tumor effects using a tumor-bearing mouse model and normal controls. Local administration of ozonated water (208 mM) was not associated with any detrimental effects in normal tissues. On the other hand, local administration of ozonated water (20.8, 41.6, 104, or 208 mM) directly into the tumor tissue induced necrosis and inhibited proliferation of tumor cells. There was no significant difference in the number of terminal deoxynucleotidyl transferase-mediated deoxyuridine triphosphate-biotin nick-end labeling (TUNEL)-positive cells following administration of ozonated water. The size of the necrotic areas was dependent on the concentration of ozonated water. These results indicate that ozonated water does not affect normal tissue and damages only the tumor tissue by selectively inducing necrosis. There is a possibility that it exerts through the production of reaction oxygen species (ROS). In addition, the induction of necrosis rather than apoptosis is very useful in tumor immunity. Based on these results, we believe that administration of ozonated water is a safe and potentially simple adjunct or alternative to existing antineoplastic treatments.

## 1. Introduction

Ozone is an active form of oxygen consisting of three oxygen atoms that is generated from diatomic oxygen by ultraviolet light and high voltage. Ozone gas is clear, colorless, and smells slightly like grass. Moreover, it is very unstable and decomposes into diatomic oxygen within a few hours in air, and has a very short half-life of approximately 30 min in water [1]. Ozone therapy has been receiving increasing attention in Europe in recent years. In this regard, major autohemotherapy (MAH), the main form of ozone therapy, is known for its beneficial effects as adjunct therapy for cardiovascular disease, infection, cancer, rheumatoid arthritis, and osteoarthritis [2]. In MAH, the patient’s blood is mixed with ozone gas before being retransfused. A previous report indicated that MAH in patients with cancer may improve tumor hypoxia and enhance sensitivity to both chemotherapy and radiation therapy [3]. In a clinical setting, a combination of radiation therapy and MAH or rectal administration of an ozone/oxygen gas mixture achieved the same effects as a combination of radiation therapy and chemotherapy in preventing progression of head and neck cancer [4]. However, there have been no reports on the direct effects of ozone gas on tumor cells *in vivo*, and to the best of our knowledge, only the *in vitro* effects have been studied [5,6].

While ozone gas therapy has many advantages, it has not been widely used in medical settings since ozone gas is toxic to the respiratory tract of the lung [7,8]. In contrast, ozonated water which means the water dissolved ozone gas has no reported side effects, and it is much easier to handle than ozone gas. Despite these advantages, however, ozonated water has not found widespread application in medicine because it is difficult to obtain an exact concentration of ozone in water. Recently, a device has been developed that can produce ozonated water to an exact concentration [9]. We made use of this device to study the safety and anti-tumor effects of ozonated water through the administration of ozonated water to normal and tumor-bearing mice.

## 2. Results and Discussion

### 2.1. Effects of Administration of Ozonated Water into Normal Tissue

**Figure 1:**
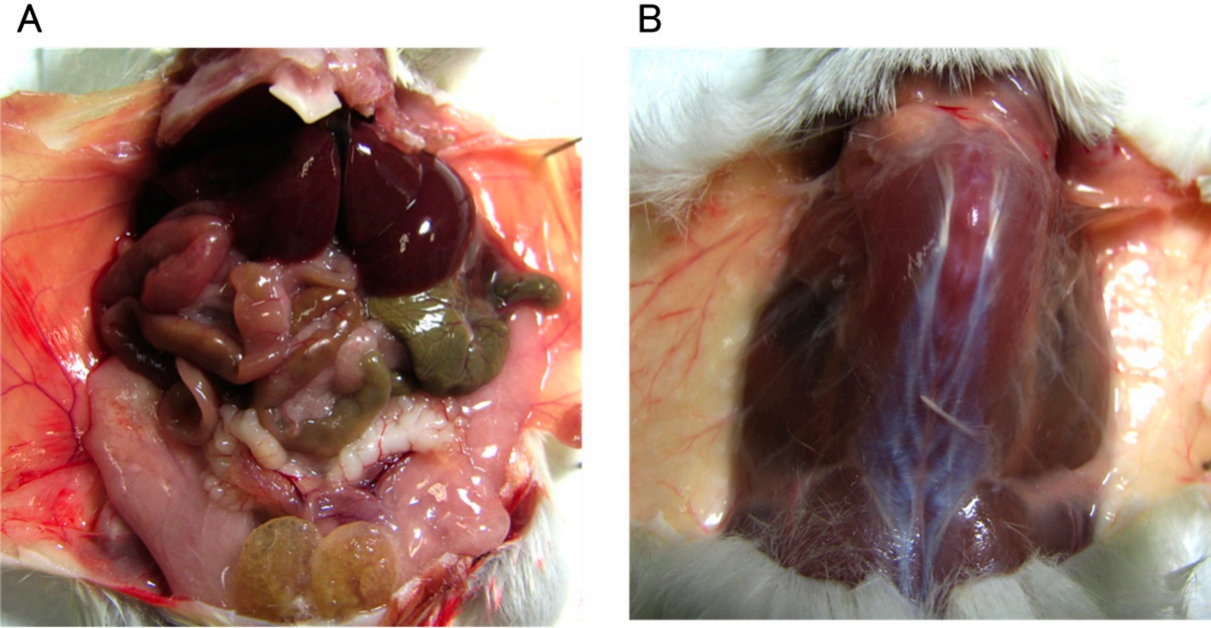
Macroscopic images of the organs in the abdominal cavity (**A**) and subcutaneous tissue (**B**).

**Figure 2:**
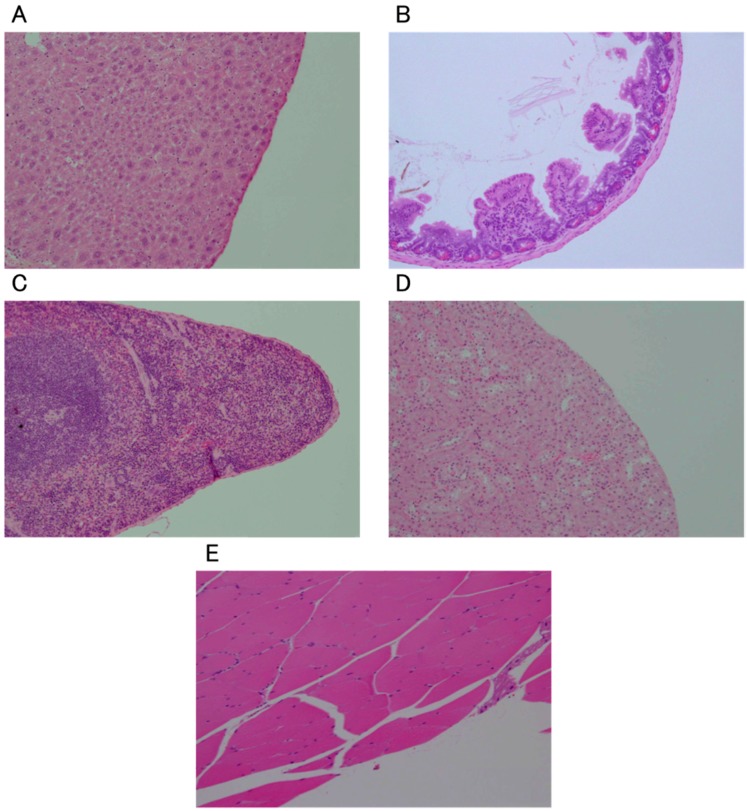
Histological images of normal tissues of the liver (**A**), small intestine (**B**), spleen (**C**), kidney (**D**) and muscle (**E**) 24 h after the last administration of ozonated water. All sections were observed (100×) after staining with hematoxylin and eosin.

In the intraperitoneal administration (IP) group, no abnormalities such as edema of the organs or ascites were observed macroscopically (Figure 1A). No abnormalities were observed in any of the examined organs (liver, spleen, kidney, and small intestine) on histopathological examination (Figure 2A–D). In the subcutaneous administration (SC) group, no subcutaneous abnormalities were observed macroscopically (Figure 1B). In the intramuscular administration (IM) group, no abnormalities such as claudication were observed in any mice. No abnormalities were observed on histopathological examination either (Figure 2E).

### 2.2. Concentration and Time-Dependent Effects of Ozonated Water after Local Injection in Tumor Tissue

**Figure 3:**
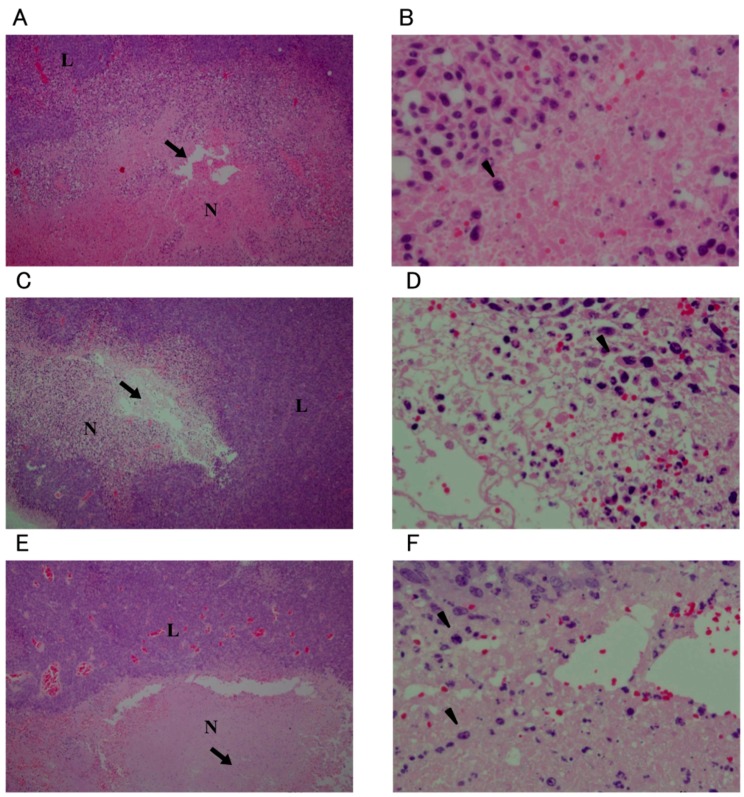
Histological images of tumors 1 day after administration of 20.8, 41.6, or 104 mM ozonated water. Low magnification (40×): (**A**,**C**,**E**); High magnification (400×): (**B**,**D**,**F**). The concentration of ozonated water was 20.8 mM (**A**,**B**); 41.6 mM (**C**,**D**); or 104 mM (**E**,**F**). All sections were stained with hematoxylin and eosin. N: necrotic area; L: viable tumor area; arrow: site of injection; arrowhead: pyknotic nuclei.

**Figure 4:**
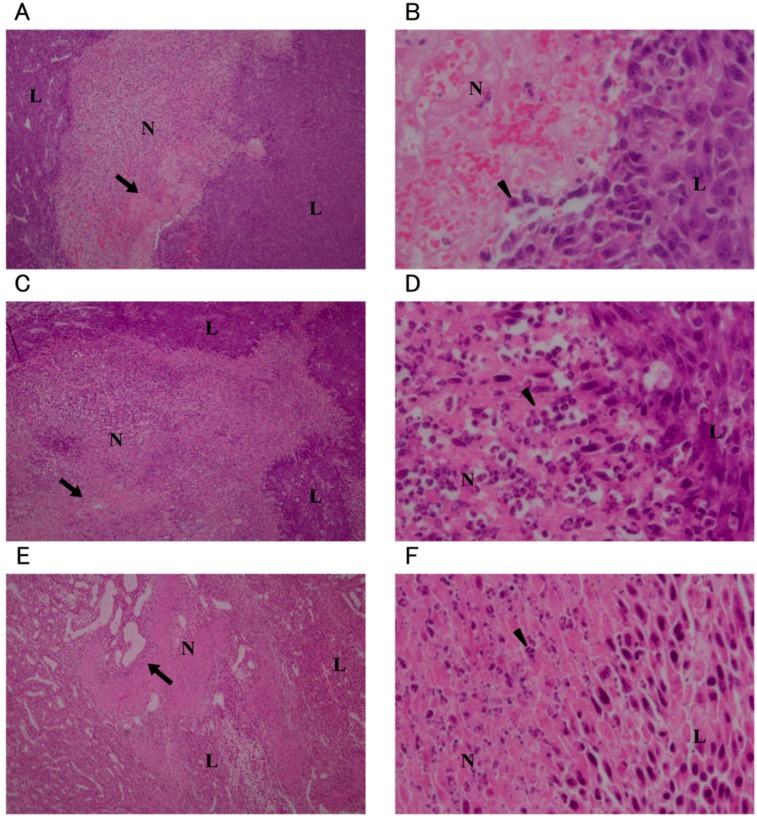
Histological images of tumors after administration of 208 mM ozonated water. the O_3_-4/1 day group is (**A**,**B**); O_3_-4/3 day group is (**C**,**D**); O_3_-4/7 day group is (**E**,**F**). Low magnification (40×): (**A**,**C**,**E**); High magnification (400×): (**B**,**D**,**F**). All sections were stained with hematoxylin and eosin. N: necrotic area; L: viable tumor area; arrow: site of injection; arrowhead: pyknotic nuclei.

**Figure 5:**
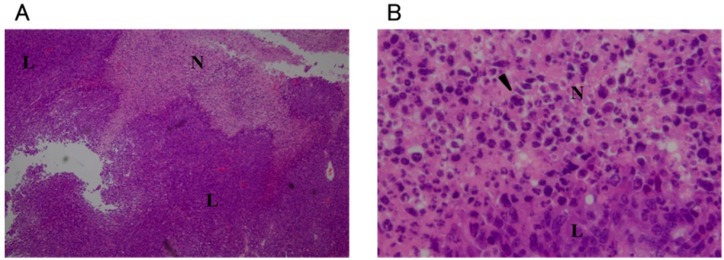

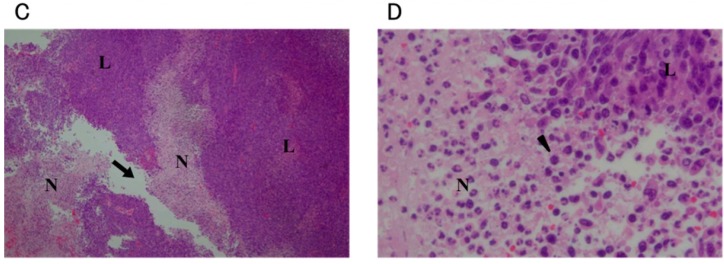
Histological images of tumors that were either untreated or administered sterile distilled water, after 24 h. Non-treated (**A**,**B**); treated with sterile distilled water (**C**,**D**). Low magnification (40×): (**A**,**C**); High magnification (400×): (**B**,**D**). All sections were stained with hematoxylin and eosin. N: necrotic area; L: viable tumor area; arrow: site of injection; arrowhead: pyknotic nuclei.

Necrosis was observed around the injection site in all groups except for the non-treatment (NT) group. In the O_3_-1, O_3_-2, and O_3_-3 groups, nuclei were not observed in the tumor cells in the centermost areas of necrosis. Pyknotic nuclei were abundant at the edges of the necrotic region (Figure 3). In the O_3_-3 group, the area of necrosis was particularly broad and the area in which nuclei were not observed in the tumor cells was larger compared to the O_3_-1 and O_3_-2 groups (Figure 3E). In the O_3_-4/1 day, O_3_-4/3 day, and O_3_-4/7 day groups, necrosis in the tumor tissues was observed to about the same extent as that in the other ozonated water groups (Figure 4). However, viable tumor cells were observed in the necrotic area in the O_3_-4/7 day group (Figure 4E). In the NT and local administration of sterile distilled water (solvent control) into the tumor tissue (C) groups, many pyknotic nuclei were seen in the necrotic areas (Figure 5). In the NT group, pyknotic nuclei were more frequent in the centermost regions of tumor tissue compared with other groups (Figure 5A,B).

### 2.3. The Effects of Local Administration of Ozonated Water on Tumor Growth

**Figure 6:**
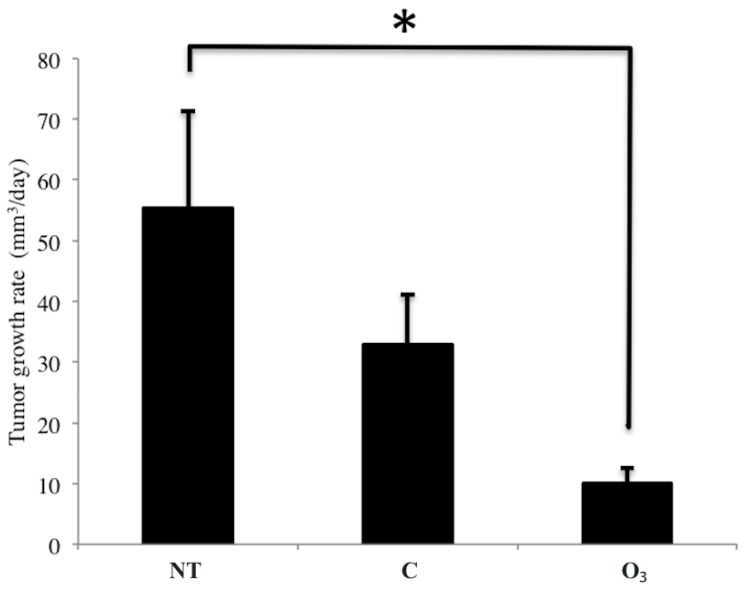
The effect of local administration of ozonated water on rates of tumor growth. The data represent the mean ± SE; *n* = 5 in the NT and C groups, *n* = 6 in the O_3_ group. * *p* < 0.05 compared to the NT group by using the Tukey–Kramer test. NT: non-treatment group; C: local administration of sterile distilled water group; O_3_: local administration of ozonated water group.

The tumor growth rate in the O_3_ group (9.9 ± 2.7 mm^3^/day) was significantly decreased compared to that of the NT group (55.3 ± 16.0 mm^3^/day) (*p* < 0.05, Figure 6). The tumor growth rate in the C group was 32.9 ± 8.1 mm^3^/day. No significant difference was seen between the O_3_ and C groups. However, a tendency was observed toward decreased rates of tumor growth in the O_3_ group compared to the C group.

**Figure 7:**
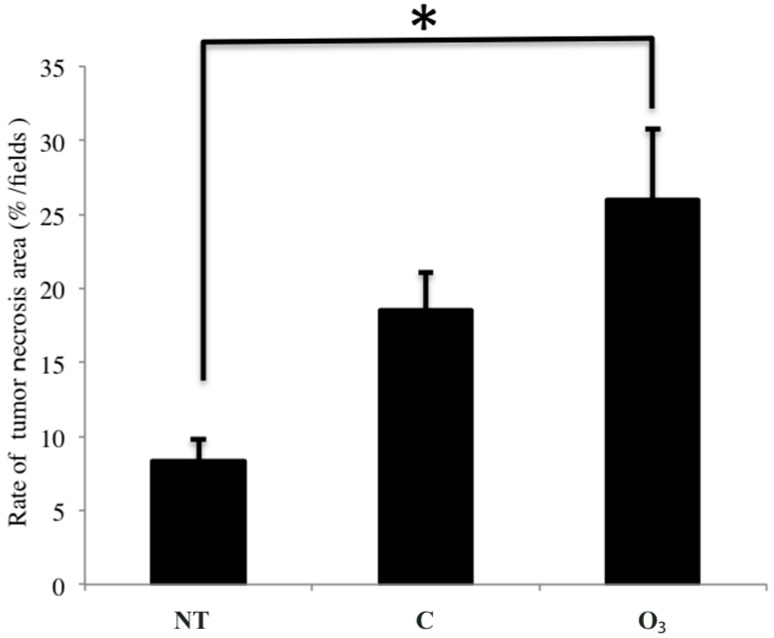
The effect of local administration of ozonated water on size of areas of tumor necrosis. The data represent the mean ± SE; *n* = 5 in the NT and C groups, *n* = 6 in the O_3_ group. * *p* < 0.05 compared to the NT group by using the Tukey–Kramer test. NT: non-treatment group; C: local administration of sterile distilled water group; O_3_: local administration of ozonated water group.

The area of tumor necrosis was significantly increased in the O_3_ group ((26.0% ± 5.2%)/field), compared to that in the NT group ((8.3% ± 1.5%)/field) (*p* < 0.05, Figure 7). Meanwhile, the area of tumor necrosis in the C group was (18.5% ± 2.6%)/field, with no significant difference from that in the O_3_ group. However, a tendency toward increased tumor necrosis was seen in the O_3_ group compared to the C group. The number of TUNEL-positive cells in the three groups did not significantly differ (Table 1).

**Table 1:** The number of erminal deoxynucleotidyl transferase-mediated deoxyuridine triphosphate-biotin nick-end labeling (TUNEL) positive cells in tumors.

Staining	NT	C	O_3_
TUNEL positive cells (cells/field)	7.92 ± 0.63	9.24 ± 0.61	9.34 ± 1.02

The data represent the mean ± SE; *n* = 5 in the NT and C groups, *n* = 6 in the O_3_ group. There was no significant difference between the three groups. NT: non-treatment group; C: local administration of sterile distilled water group; O_3_: local administration of ozonated water group.

### 2.4. Discussion

It has been shown that ozonated water has antibacterial effects [10,11]. Ozmen *et al.* reported that “ozonated saline was effective as irrigation for treating experimental peritonitis rats” [12]. To the best of our knowledge, the side-effects of ozonated water have not yet been reported. In the present study, it was found that ozonated water did not affect normal tissue. Moreover, ozonated water was found to inhibit tumor growth through the induction of necrosis of tumor cells. During the experimented period, no detrimental effects were observed following local administration of ozonated water. These results indicate that administration of ozonated water has low toxicity and is safe and very useful in anti-tumor treatment. However, these results are only indicative of a short-term effect. Therefore, further information regarding the potential long-term effects of ozonated water is needed.

Necrosis of the tumor cells was observed following local injection of ozonated water directly into the tumor tissues. Moreover, the tumor growth rate was significantly decreased and there was a significant increase in the tumor necrotic area. However, there were no significant differences among the three groups in the number of TUNEL-positive cells. These results indicate that local administration of ozonated water does not induce apoptosis, although it does act directly on the tumor cells. It has been reported that ozone gas directly inhibits neoplastic cell growth in the neuroblastoma SK-N-SH cell line *in vitro* by modulating the cell cycle [6]. However, to the best of our knowledge, no previous studies have investigated the direct effects of ozone on tumor tissue *in vivo*. This report is the first regarding the direct effects of ozone on tumor tissue.

In this study, the tendency that the tumor growth rate is decreased and the area of tumor necrosis is increased was observed in C group. However, these data were not significantly compared to that in NT group. We think that it was induced by physical injury when water and/or osmotic pressure was injected.

Many tumor cells have few antioxidant materials, such as superoxide dismutase (SOD), catalase, and glutathione peroxidase, in comparison with normal cells [13]. Therefore, the tumor cells are greatly affected by reactive oxygen species (ROS). It is well known that in a variety of anticancer agents, such as adriamycin and *cis*-diamminedichloroplatinum (II), ROS play important roles in the cytotoxic effects on tumor cells [14,15]. High-dose ascorbic acid therapy controls the growth of aggressive tumors by producing ROS [16]. ROS can interact with numerous cellular components, including DNA, lipids, and proteins [17]. The consequences of these interactions include losses of cell integrity, enzyme function, and genomic stability [18,19]. When ozone is administered *in vivo*, it is well known that ROS such as hydrogen peroxide (H_2_O_2_) are generated [20]. Therefore, the anti-tumor mechanism of ozonated water is likely to also be the cell injury caused by ROS. It will be necessary to verify the association between ROS and anti-tumor effects of ozonated water using ROS inhibitors.

The necrotic cell releases danger signals such as deoxyribonucleic acid (DNA), high mobility group box 1 protein (HMGB1), and uric acid [21,22,23]. On the other hand, the apoptotic cell does not release these signals [22,24]. An antigen-presenting cell (APC), particularly the dendritic cell (DC), matures following stimulation by cytokines and danger signals, after uptake of the tumor antigen. In conditions in which danger signals are lacking, APCs cannot mature and activate T cells. Therefore, the growth of tumors may be promoted under the non-existence of danger signals. In a previous report, a decrease in metastasis and an extension in the duration of survival were observed in a mouse tumor necrosis model compared to a mouse tumor apoptosis model [25]. Therefore, the induction of necrosis rather than apoptosis by administration of ozonated water is very useful in tumor immunity.

It has been reported that the production of several cytokines including interferon (IFN)-α, IFN-β, and IFN-γ, interleukin (IL)-1, IL-2, tumor necrosis factor (TNF)-α, and granulocyte-macrophage colony-stimulating factor was induced after human blood, heparinized platelet-rich plasma, and Ficoll-purified blood mononuclear cells were ozonated [26,27,28,29]. Previous reports have indicated that cytokines inhibit both tumor growth and metastasis [30,31,32,33,34]. Therefore, cytokines may also participate in the growth restraint of tumors. Further studies on the kinetics of these cytokines following administration of ozonated water will be needed to determine their roles in the antineoplastic activity of ozonated water.

Importantly, the size of the necrotic area in the current study was dependent on the concentration of the ozonated water. Concentration-dependent growth inhibition has been reported *in vitro* in human cancer cells from lung, breast, and uterine tumors following treatment with ozone gas [5]. Herein, when the observation time was extended, viable tumor cells were observed in the necrotic area in the O_3_-4/168 h group. This result indicates that a single administration of ozonated water cannot achieve complete destruction of tumor cells, and thus repeated administration is required.

## 3. Experimental Section

### 3.1. Preparation of Ozonated Water

The device producing ozonated water was provided by Sakuragawa Pump Co., Ltd., (Osaka, Japan). This device can produce ozonated water from O_2_ and water and can regulate the concentration of the dissolved O_3_. In all experiments, ozonated water was administered within 10 min after production.

### 3.2. Animals

BALB/c mice (4–5 week-old, female) were purchased from CLEA Japan, Inc. (Osaka, Japan). All mice were maintained under conventional conditions. The study was carried out according to Tottori University animal experiment rule. The use of these animals and the procedures undertaken were approved by the Animal Research Committee of Tottori University (Permit Number: 13T4). All treatments were performed under anesthesia induced by inhalation of 3%–5% isoflurane and all efforts were made to minimize suffering.

### 3.3. Preparation of the Tumor-Bearing Mouse Model

Mouse cell line derived from mouse rectal cancer, Colon 26 (RCB2657), was obtained from by the RIKEN BioResource Center (BRC) through the National Bio-Resource Project of the MEXT (Ibaraki, Japan). The tumor-bearing mouse model was prepared as previously described, with slight modification [35]. In brief, 1 × 10^6^ colon 26 cells (1 × 10^7^ cells/mL) were subcutaneously injected into the dorsal regions in BALB/c mice. Mice whose tumors grew to a diameter of 7–10 mm were used in the experiments.

### 3.4. Effects of Administration of Ozonated Water into Normal Tissue

Mice were randomized into three groups: intraperitoneal administration (IP) group, subcutaneous administration (SC) group, and intramuscular administration (IM) group. In the IP and SC groups, 1 mL of 208 µM ozonated water was administered under anesthesia induced by inhalation of 3%–5% isoflurane. In the IM group, 0.1 mL of 208 mM ozonated water was administered. Ozonated water was administered for three days. Twenty-four hours after the last administration, all mice were euthanized by cervical dislocation under anesthesia induced by inhalation of 3%–5% isoflurane. Each organ, as well as samples of subcutaneous tissue and muscle, were observed macroscopically and fixed in 10% buffered formalin.

### 3.5. Concentration and Time-Dependent Effects of Ozonated Water after Local Injection in Tumor Tissue

Mice were randomized into six groups: non-treatment (NT) group; local administration of sterile distilled water (solvent control) into the tumor tissue (C); local administration of 20.8 mM ozonated water into the tumor tissue (O_3_-1); local administration of 41.6 mM ozonated water into the tumor tissue (O_3_-2); local administration of 104 mM ozonated water into the tumor tissue (O_3_-3) (*n* = 3 in each group); and local administration of 208 mM ozonated water into the tumor tissue (O_3_-4) (*n* = 9). One milliliter of sterile distilled or ozonated water was administered under anesthesia induced by inhalation of 3%–5% isoflurane. In the NT, C, O_3_-1, O_3_-2, and O_3_-3 groups, all mice were euthanized by cervical dislocation and the tumors were resected 24 h after administration. In the O_3_-4 group, all mice were euthanized by cervical dislocation and the tumors were resected one, three, and seven days after administration (O_3_-4/1 day, O_3_-4/3 day and O_3_-4/7 day groups, each *n* = 3). The tumors were fixed in 10% buffered formalin.

### 3.6. The Effects of Local Administration of Ozonated Water on Tumor Growth

Mice were randomized into three groups: NT group (*n* = 5); local administration of sterile distilled water (solvent control) into the tumor tissue (C, *n* = 5); and local administration of ozonated water into the tumor tissue (O_3_, *n* = 6). The volumes of the tumor tissues were calculated as follows: (mediastinum × transverse line × depth × π)/6 (mm^3^). After calculations of the tumor volumes, distilled water or 208 mM of ozonated water was locally administered into the tumor tissues (0.2 mL/head, day 1). After three days (day 4), a second local injection of distilled water or 208 mM ozonated water was given. On day 7, mice were euthanized by cervical dislocation under anesthesia induced by inhalation of 3%–5% isoflurane. Based on the tumor volumes on days 1 and 7, the tumor growth rates were calculated as follows: (tumor volume on day 7 − tumor volume on day 1)/7 (mm^3^/day). The tumors were fixed in 10% buffered formalin.

### 3.7. Histological Evaluation and Image Analysis

Thin sections (5 μm) were cut from each sample for histological observation after staining with hematoxylin and eosin. Each section was examined microscopically and quantitative digital morphometric analysis of necrotic areas was performed. The color wavelengths of the copied images were transformed into digital readings using the Lumina Vision software program (Mitani Corporation, Tokyo, Japan), which allows for quantification of the various color wavelengths with pixels as the unit of measurement. The percentage of necrosis in the tumor tissues was calculated by dividing the total pixel area of the necrotic area by the total pixel area corresponding to the total tumor tissue in the field of view [35].

Apoptosis of the tumor tissues was investigated using terminal deoxynucleotidyl transferase-mediated deoxyuridine triphosphate-biotin nick-end labeling (TUNEL) staining. Staining was performed according to previously described methods [36]. Each section was examined microscopically and the number of TUNEL-positive cells in each tumor was calculated. Cell counts were calculated in five fields, excluding necrotic regions, at 400× magnification using five mice in each group. The mean score of 25 fields was considered the number of TUNEL-positive cells in each group.

### 3.8. Statistical Analysis

Data were expressed as the mean ± standard error (SE). Statistical analyses were first performed using one-way analysis of variance (ANOVA) and followed and compared using the Tukey–Kramer test. A value of *p* < 0.05 was considered statistically significant.

## 4. Conclusions

In this study, it is suggested that ozonated water has an anti-tumor effect. There is a possibility that it is exerted through the production of ROS. Moreover, ozonated water has low toxicity and is safe and easy to handle. On the other hand, ozone gas is dangerous and not easy to handle. This is because ozone gas is toxic to the respiratory tract of the lung [7,8]. Administration of ozonated water is a safe and potentially simple adjunct or alternative to existing antineoplastic treatments.
